# Influence of Temperature on the Life-Cycle Dynamics of *Aedes albopictus* Population Established at Temperate Latitudes: A Laboratory Experiment

**DOI:** 10.3390/insects11110808

**Published:** 2020-11-17

**Authors:** Giovanni Marini, Mattia Manica, Daniele Arnoldi, Enrico Inama, Roberto Rosà, Annapaola Rizzoli

**Affiliations:** 1 Research and Innovation Centre, Department of Biodiversity and Molecular Ecology, Fondazione Edmund Mach, 38010 San Michele all’Adige, Italy; mattia.manica@protonmail.ch (M.M.); daniele.arnoldi@fmach.it (D.A.); enrico.inama@fmach.it (E.I.); roberto.rosa@unitn.it (R.R.); annapaola.rizzoli@fmach.it (A.R.); 2 Epilab-JRU, FEM-FBK Joint Research Unit, Province of Trento, 38100 Trento, Italy; 3 Center for Information and Communication Technology, Bruno Kessler Foundation, 38123 Trento, Italy; 4 Center Agriculture Food Environment, University of Trento, 38010 San Michele all’Adige, Italy

**Keywords:** mosquito bionomics, mosquito dynamics, invasive species

## Abstract

**Simple Summary:**

Mosquitoes represent a potential major public health concern, as they are capable of transmitting several pathogens when biting humans. It is well known that temperature is a crucial factor affecting mosquito biology: for instance, warmer conditions can increase survival and fecundity. Here, we quantify the influence of different temperatures on the bionomics of *Aedes albopictus*, which is a mosquito species native to Southeast Asia that has been able to spread worldwide during the last forty years. We used specimens collected from northern Italy to assess if temperate individuals are characterized, possibly thanks to an adaptation process, by a different thermal response with respect to subtropical individuals. We found that immature stages are well adapted to colder temperatures, which nonetheless seem to prevent any blood-feeding activity. Adult longevity and fecundity were substantially greater at mild conditions. This thermal adaptation might increase the length of the breeding season and could allow the colonization of areas at higher altitude, resulting in an overall increased risk for potential transmission of *Ae. albopictus*-borne pathogens.

**Abstract:**

The mosquito species *Aedes albopictus* has successfully colonized many areas at temperate latitudes, representing a major public health concern. As mosquito bionomics is critically affected by temperature, we experimentally investigated the influence of different constant rearing temperatures (10, 15, 25, and 30 °C) on the survival rates, fecundity, and developmental times of different life stages of *Ae. albopictus* using a laboratory colony established from specimens collected in northern Italy. We compared our results with previously published data obtained with subtropical populations. We found that temperate *Ae. albopictus* immature stages are better adapted to colder temperatures: temperate larvae were able to develop even at 10 °C and at 15 °C, larval survivorship was comparable to the one observed at warmer conditions. Nonetheless, at these lower temperatures, we did not observe any blood-feeding activity. Adult longevity and fecundity were substantially greater at 25 °C with respect to the other tested temperatures. Our findings highlight the ability of *Ae. albopictus* to quickly adapt to colder environments and provide new important insights on the bionomics of this species at temperate latitudes.

## 1. Introduction

*Aedes albopictus* is a mosquito species native to Southeast Asia that has been able to spread worldwide during the last forty years. In particular, it has successfully colonized many areas in several European countries, and it was the subject of European guidelines to contain and prevent its further spread [1,2]. Nowadays, its distribution in Europe is constantly monitored, and updated distribution maps are available [3]. *Aedes albopictus* represents a major public health concern in all countries where it has established since it is a competent vector of a broad range of viruses such as Zika (ZIKV), dengue (DENV), and chikungunya (CHIKV) [4,5]. The *Ae. albopictus* mosquito was responsible for the two largest European chikungunya outbreaks that both occurred in Italy in 2007 and 2017 [6,7], and it has been associated with the autochthonous transmission of ZIKV, CHIKV, and DENV in Croatia, France, Italy, and Spain [8,9,10,11,12,13].

Previous modeling studies predicted a further spread of *Ae. albopictus* in new areas [14,15,16,17], which has been confirmed by recent observations. Usually, temperature is one of the key components of such model predictions, as mosquito population dynamics (e.g., survival, development, fecundity) are critically affected by environmental temperatures [18,19,20,21,22]. Therefore, quantitative information on this relationship is crucial to assess the population dynamics and the potential environmental suitability for different mosquito species under climate change scenarios. However, despite their relevance in terms of rapid risk assessment for public health, quantitative estimate of such parameters under different environmental and climatic conditions are often lacking.

Several laboratory studies have been carried out to quantify the effect of different temperatures on *Ae. albopictus* development and survival [18,23,24,25], showing that warmer conditions are in general associated with shorter developmental times and very high temperatures (e.g., 35 °C) result in a shorter adult longevity. In addition, field experiments showed a positive correlation between abundance and temperature, suggesting that warmer temperatures may shorten developmental time or improve survival and favor mosquito spread [26,27]. However, *Ae. albopictus* has showed strong ecological plasticity and may in the future well adapt to colder than expected climates [28].

The study carried out by Delatte and coauthors [18] is to the best of our knowledge one of the most recent and widely referred as a source for parameter values in *Ae. albopictus* modeling (e.g., [29,30,31,32,33,34,35]). In their study, the authors investigated *Ae. albopictus* immature stages survival and development, adult longevity, and fecundity at different constant temperatures, ranging from 5 to 40 °C, using specimens from a laboratory colony established from field-collected mosquitoes in La Réunion Island, France (55°29′ S and 47°51′ W), which is an area characterized by a subtropical climate.

Thus, we performed further experiments to investigate whether *Ae. albopictus*, collected from a temperate area where it has established for more than a decade, is characterized, possibly thanks to an adaptation process, by different life traits with respect to subtropical individuals. To this aim, we replicated Delatte’s experiments for a subset of temperatures using specimens from the province of Trento (northern Italy) where *Ae. albopictus* has been present since at least 1996 [36,37].

## 2. Materials and Methods

### 2.1. Mosquito Colony

A colony of *Ae. albopictus* was established in 2018 in the insectary of Fondazione Edmund Mach (San Michele all’Adige, Italy) from eggs collected in the field in the province of Trento (Italy). Adults were kept in a cage 45 × 45 × 45 cm (Bugdorm, MegaView Science Co., Ltd., Taiwan) in a climatic room at 25 ± 1 °C and a relative humidity (RH) of 75 ± 5%, with a photoperiod of 16L:8D with 1 h of dawn and 1 h of dusk. The colony was supplied with cotton soaked in 10% common white sugar (sucrose) solution ad libitum. Twice a week, the colony was fed on cow blood for 1 h provided with a Hemotek blood-feeding system (Hemotek Ltd., Accrington, England). Two ovitraps lined with filter paper along the inner side as an oviposition substrate were placed inside the cage. Then, strips with eggs were removed and placed in plastic cups, stored in plastic bags, loosely sealed, and sprayed with water every week to maintain high RH.

### 2.2. Experimental Conditions

Environmental chambers (Proclimatic Lt 900 ACLI 4000, Imola, Italy) were used to carry out the experiments. They were programmed with a photoperiod of 12:12 (L:D) with 1 h of dawn and 1 h of dusk under four constant temperatures (10 ± 0.5, 15 ± 0.5, 25 ± 0.5 and 30 ± 0.5 °C). During all experiments, RH was controlled and fixed at 80 ± 3%, as in [18].

### 2.3. Egg-Hatching Experiment

We followed a protocol similar to the one proposed in [18]. Filter paper was collected from the colony cage within 1–2 days after placement so that eggs were only a few days different in age. Eggs of 5–9 days-old (incubated at 25° and 80% RH) were placed in a group of 10 in cups (12 replicates) filled with 30 mL of dechlorinated water. No food was added as hatching stimuli. Cups were randomly rotated to prevent any positional effect. The 10-eggs papers were left in water for 5 days and larvae were counted daily and then removed. Every 5 days, eggs were taken out of the water and dried for 2 days. After three times, unhatched eggs were regarded as nonviable.

### 2.4. Larval Survival and Developmental Time

We followed a protocol similar to the one proposed in [18]. Eggs were placed in water for hatching at 25 °C, and subsequently, larvae less than 2-h-old were isolated in groups of 10 using small pipettes in cups filled with 120 mL of dechlorinated water. For each temperature, ten repetitions of 10 larvae each were monitored. Cups were randomly rotated to prevent any positional effect. Every day, exuviae and dead larvae were removed. The stage of each living larva was identified, and the number of each instar was counted. Larvae were fed daily with brewers yeast (LIEVITAL-Lesaffre Italia, Italy), with the quantity of food increasing according to the stage of development (0.2 mg of yeast per L1, 0.4 mg per L2, 0.6 mg per L3, and 0.8 mg per L4). At the beginning of pupation, pupae were isolated, and then the number of male and female adults was counted.

### 2.5. Adult Longevity and Gonotrophic Cycle

Larvae were reared at 25 °C and 80% RH in 500 mL plastic cups filled with 250 mL of dechlorinated water and were fed daily following the protocol described above. After emergence, adult mosquitoes (males and females) of the same age (less than 12-h old) were isolated in a 22 × 22 × 22 cm cage with 10% common white sugar solution ad libitum and left together 3–4 days for free mating. After this period, females were allowed to feed on cow blood provided by Hemotek blood-feeding system. Then, one male and one engorged female of the same age were isolated as a couple in a cage with an ovitrap filled with 250 mL of dechlorinated water, added with 50 mL of grass infusion, and lined with filter paper as oviposition substrate. A piece of cotton, soaked with 10% sugar solution, was provided to each cage and replaced twice a week. Twice a week, cow blood was offered to each female by Hemotek and, immediately after, every female was observed to see if they were fed. The presence of laid eggs on the filter paper and dead male and/or female were checked daily. Eggs, if present, were counted and removed. Experiments were carried out with 20 couples for each tested temperature (10, 15, 25, and 30 °C).

### 2.6. Statistical Analysis

We considered the following quantities: (i) survival and (ii) developmental times of eggs and immature stages (four larval instars and pupa); (iii) female longevity, (iv) number of eggs laid by an adult female, (v) number of gonotrophic cycles and (vi) gonotrophic cycle length. We compared all these quantities at different tested temperatures. We applied a Bayesian approach by fitting through a Markov Chain Monte Carlo technique [38] a Poisson distribution for developmental times, longevity, number of laid eggs, number and length of the gonotrophic cycles, and a binomial distribution for survival rates. Then, we sampled 10,000 values from the posterior distribution of the parameter of interest (for instance, the mean duration of development from L1 to A) for two tested temperatures, computed the differences between the two distributions, and assessed the probability of drawing zero from the distribution of differences. Finally, we analyzed adult survival using a Weibull model (age-dependent survival), as in [18]. Then, we stochastically simulated the population outcome of 1000 initial eggs at two different temperatures (25 and 30 °C) to assess the expected number of emerging adults, time of development, adult longevity, and fecundity. Additional methodological details and results, including histograms of the estimated posterior distributions, are reported in Appendix A.

We also tested whether our observations differed significantly from the ones for subtropical *Ae. albopictus* reported in [18]. Specifically, for survival rates, developmental times, number of laid eggs, and number and length of the gonotrophic cycles, we sampled 10,000 values from the posterior distribution of the parameter of interest (for instance, the mean duration of development from L1 to A at a specific temperature) and evaluated whether the averages presented for subtropical *Ae. albopictus* in [18] lie in the 95% Confidence Intervals (CI) of the estimated posterior distributions.

## 3. Results

### 3.1. Survivorship and Length of Development of Eggs and Immature Stages

**Figure 1:**
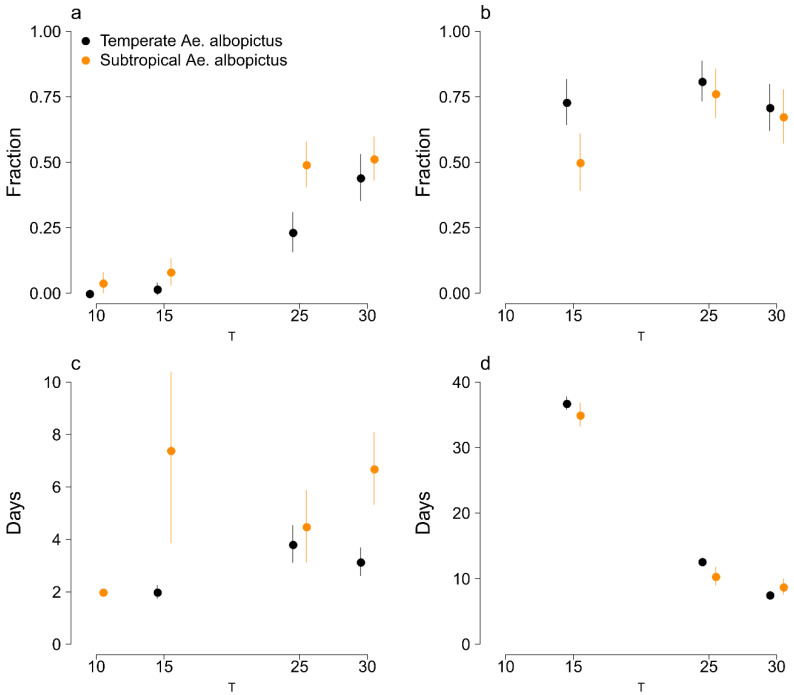
Comparison between temperate *Ae. albopictus* (black) and subtropical *Ae. albopictus* (orange) results for each tested temperature (10, 15, 25, and 20 °C). (**a**): Fraction of hatched eggs; (**b**): fraction of L1 larvae that successfully reached the adult stage; (**c**): length of time between immersion of eggs in water and hatching response; (**d**): duration of development from L1 to adult. Points: average values. Vertical lines: 95% Confidence Intervals (average ±1.96∙SE).

The temperate eggs hatching rate is increasingly higher at hotter temperatures. At 10 °C, we observed no hatching activity, and no larvae reached the third instar stage, but we can note that 38% of the initial individuals passed to the second instar stage, taking on average more than one month (about 37 days). We observed a similar fraction of temperate first instar larvae developing successfully into adults at 15, 25, and 30 °C (see Table 1 and Figure 1).

We did not find statistical evidence of differences in survivorship at 30 °C between temperate and subtropical eggs (*p*-value = 0.05), while at 10, 15, and 25 °C, temperate eggs are characterized by a substantially (*p*-values <0.001) lower hatching rate with respect to subtropical ones. While at 10 °C we observed some temperate larval development, all L1 subtropical individuals died before becoming L2. Overall, at 15 °C, 73% of the initial temperate larvae became adults, while for subtropical *Ae. Albopictus*, this percentage was significantly lower (50%, *p*-value <0.001) (see Table 1 and Figure 1).

There was no statistical evidence of substantial differences in the observed length of time between the immersion of eggs in water and hatching response at different temperatures. Conversely, the duration of development from L1 to adult is significantly different (*p*-values <0.001), ranging on average from more than a month (35 days) at 15 °C to about one week at 30 °C (see Table 2 and Figure 1).

Temperate eggs hatch significantly faster (*p*-values <0.05) than subtropical ones at all tested temperatures. The duration of development from L1 to adult is on average significantly shorter for subtropical larvae at 15 (*p*-value <0.01) and 25 °C (*p*-value <0.001), while conversely, at 30 °C, temperate larvae take on average substantially less time (*p*-value <0.001) to become adults (see Table 2 and Figure 1).

### 3.2. Adult Life Expectancies

**Figure 2:**
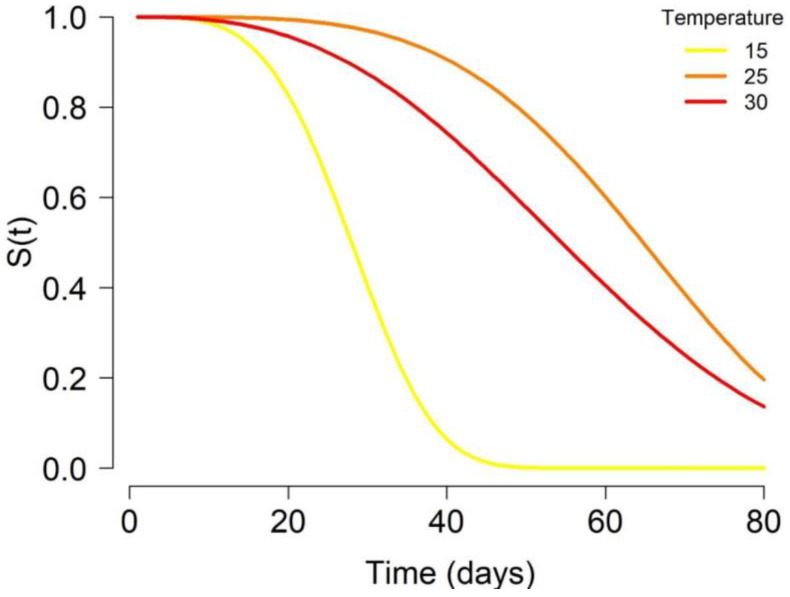
Survival rate of *Ae. albopictus* females adjusted to the Weibull’s model at temperatures of 15, 25, and 30 °C.

The average longevity of temperate female adults differs at different temperatures, being considerably shorter at low temperatures (less than a week at 10 °C). It is interesting to note that at 10 °C, all females died within 10 days, whereas at 25 °C, all individuals survived at least one month (see Table 3 and Figure 2).

Subtropical mosquitoes showed a higher survival at 30 °C than at 25 °C [18], while for temperate *Ae. Albopictus*, we found the opposite.

### 3.3. Gonotrophic Cycles and Egg Laying

At 10 °C, blood-feeding activity was not observed. At 15 °C, only two temperate females took a bloodmeal, but they did not lay any egg. At this temperature, we observed females completing the first gonotrophic cycle without taking blood (autogeny), as three individuals laid 3, 7, and 11 eggs, respectively. Conversely, at higher temperatures, all temperate females completed at least one gonotrophic cycle after taking a blood meal. Our results did not show any statistical differences between the gonotrophic cycle length at 25 and 30 °C (*p*-value >0.05), but at 25 °C, there is strong statistical evidence that substantially fewer eggs were laid (*p*-value <0.001). However, on average, a female completed 7.9 cycles at 25 °C and only 1.75 at 30 °C (Table 4).

With respect to subtropical mosquitoes [18], at 25 °C, on average, a temperate female completes a higher number of gonotrophic cycles (*p*-value <0.001), which are also significantly shorter. Conversely, at 30 °C, on average, a temperate female completes a substantially smaller number of gonotrophic cycles (*p*-value <0.001), which nonetheless last similarly to subtropical ones. Finally, at both temperatures, we found temperate mosquitoes to lay substantially fewer eggs with respect to subtropical females.

### 3.4. Population Dynamics Simulation

At 25 °C, on average, 193 adults (95%CI: 127–264) emerge from 1000 initial eggs, taking on average 16.5 days (95%CI: 9–25) under lab conditions. Conversely, at 30 °C, adults emerge faster (mean: 10.8 days, 95%CI: 5–18) and with a higher rate (mean: 311 individuals, 95%CI: 234–385), but they lay fewer eggs. In fact, at 25 °C, all emerged females are expected to lay on average about 25,000 eggs during their lifetime, while at 30 °C, this number decreases to about 15,500.

## 4. Discussion

Cold temperatures have been successful in shielding temperate area to *Ae. albopictus* establishment, in particular regarding survival between years and overwintering [14,39]. However, in our study, we found that after 20 years from invasion, temperate immature individuals have successfully adapted to colder conditions compared to subtropical populations. For instance, we found that at 15 °C, temperate eggs hatch faster. Nonetheless, 10 °C is still a lower threshold for survival, as no larval specimen survived long enough to develop into adults when kept at such temperature. This is consistent with previous findings showing that 13 °C is the temperature threshold necessary to initiate activity in adult female mosquitoes [36].

Nevertheless, it is interesting to note that at 10 °C, 38% of the first instar larvae were able to develop in L2, while at this temperature, subtropical ones did not survive beyond this stage. This capability to develop earlier in the season when temperatures are still low may increase the length of the breeding season, thus potentially impacting on the planning of control intervention and surveillance. Moreover, this adaptation could let *Ae. albopictus* colonize areas at higher altitude, where spring temperatures are generally colder with respect to lower areas. During temperate springs and summers, such low temperatures are likely to occur, even though only for a few days, but therefore, they might be not so detrimental to larval development. For instance, in the Trento municipality, the average June daily temperature between 2010 and 2019 was 20.9 °C, ranging between 12.3 and 30.1 °C. On the other hand, in this geographical area, summers are not too warm. In fact, between 2010 and 2019, only 3 days recorded an average temperature higher than 30 °C [40].

While subtropical adult average longevity was longer at 30 °C than at 25 °C, in our temperate populations we found the opposite. Another striking difference is the substantially higher fecundity at 25 °C, which is driven especially by the larger average number of gonotrophic cycles completed at such temperature. In natural conditions, this might be reflected with a greater than expected biting rate, with important public health implications. For instance, a crucial risk measure is given by the basic reproductive number R_0_, which is defined as the expected number of secondary infections that arise when a single infective host is introduced into a fully susceptible host population through pathogen transmission by the vector (see, for instance, [41]). A higher biting rate translates into a larger R_0_, thus dramatically affecting for instance the probability of occurrence of an outbreak and its final size. Nonetheless, we need to remark that our experiment protocol was not exactly identical to the one followed by Delatte and coauthors. In fact, subtropical mosquitoes were offered a blood meal from an anesthetized mouse every day, while, due to logistic constraints, we offered cow blood twice a week via Hemotek. Moreover, we should note that adult survival under laboratory rather than natural conditions may have been inflated due to the availability of food and the absence of both predation and host defensive mechanisms against blood feeding.

The developmental times of aquatic stages are important for the correct planning of larvicide interventions. The vast majority of authorized larviciding products in Europe target specific stages (for instance, first and second larval instar), and their effectiveness could last up to 3/4 weeks after treatment under common use [42]. However, a short developmental time may take advantage of any delay in larvicide application, as well as cause a selection of small temporary breeding sites that are not suitable for larvicide interventions.

The use of a lab colony, even if mosquitoes were recently collected from the field, and the experimental conditions may have inflated survival rates, for instance thanks to a lack of predation or shortened developmental times, as there was no excessive competition for resources and no temperature fluctuation compared to field condition. Indeed, constant rearing conditions, as in the laboratory, might increase mosquito longevity and fecundity. Although these are clear limitations, they are widely accepted, as the study of such quantities in field conditions is extremely difficult. To overcome these issues, an interesting modeling framework to directly estimate such parameters from field-monitoring data was recently proposed [43]. However, even if that approach does allow for the estimation of biological parameters, it adds more complexity as many factors, ranging from computational issues to availability of meteorological data or specimen dispersal, are introduced.

Possible future studies might investigate adaptation in overwintering capability. Given our results for immature and adult specimens, it is likely that diapausing temperate eggs are more resistant to colder temperatures, as it was proven in North America [44].

Several mathematical models have been developed to study *Ae. albopictus* population dynamics and the transmission of viruses such as ZIKV, CHIKV, or DENV, and most of them explicitly incorporate temperature-dependence [30,31,34,45]. Other kinds of studies aimed at identifying new areas of potential invasion, usually considering temperature as one of the main predictors [14,15,16,17,46]. Therefore, our findings might be helpful to better tailoring such models at temperate latitudes, providing new important insights on the adaptation of this species to colder climate and on the likelihood of pathogen transmission, such as ZIKV, CHIKV, or DENV.

## 5. Conclusions

*Aedes albopictus* adaptation to colder habitats may have critical consequences for European public health safety by exposing previously unaffected areas to the potential transmission of arboviruses. In this study, we provided new important evidence on how such an adaptation process is occurring in temperate conditions by compiling additional knowledge regarding the life cycle features of *Ae. albopictus*. This adaptation might increase the length of the breeding season and could allow the colonization of areas at higher altitude, resulting in an overall increased risk for the potential transmission of *Ae. albopictus*-borne pathogens.

## Figures and Tables

**Table 1:** Temperate *Ae. albopictus* immature survival. Survival rate (as fraction), with standard error, from hatching to emergence of temperate (TE) and subtropical (ST) *Ae. albopictus* maintained at four constant temperatures: 10, 15, 25, and 30 °C.

T (°C)	Eggs (n)		Egg-L1		L1 (n)		L1-L2		L2-L3		L3-L4		L4-Pupae		Pupae-Adult		L1-Adult	
	TE	ST	TE	ST	TE	ST	TE	ST	TE	ST	TE	ST	TE	ST	TE	ST	TE	ST
10	120	100	0	0.04 ± 0.02	100	80	0.38 ± 0.05	0	0	0	0	0	0	0	0	0	0	0
15	120	110	0.02 ± 0.01	0.08 ± 0.03	100	80	0.98 ± 0.01	0.89 ± 0.04	0.99 ± 0.01	0.93 ± 0.03	1	0.86 ± 004	0.98 ± 0.01	0.84 ± 0.05	0.77 ± 0.04	0.83 ± 0.05	0.73 ± 0.04	0.5 ± 0.06
25	120	130	0.23 ± 0.04	0.49 ± 0.04	100	80	0.97 ± 0.02	0.93 ± 0.03	0.99 ± 0.01	0.95 ± 0.03	0.99 ± 0.01	0.96 ± 0.02	0.99 ± 0.01	0.97 ± 0.02	0.86 ± 0.04	0.94 ± 0.03	0.81 ± 0.04	0.76 ± 0.05
30	120	140	0.44 ± 0.05	0.51 ± 0.04	100	80	0.97 ± 0.02	0.88 ± 0.04	0.99 ± 0.01	0.99 ± 0.01	0.98 ± 0.01	0.96 ± 0.02	0.9 ± 0.03	0.91 ± 0.04	0.84 ± 0.04	0.9 ± 0.04	0.71 ± 0.05	0.68 ± 0.05

**Table 2:** Temperate *Ae. albopictus* developmental time. Length of time between immersion of eggs in water and hatching response and duration of development (mean and standard error in days) of each stage of temperate (TE) and subtropical (ST) *Ae. albopictus* maintained at four constant temperatures: 10, 15, 25, and 30 °C.

T (°C)	Eggs-L1		L1-L2		L2-L3		L3-L4		L4-Pupae		Pupae-Adult		L1-Adult	
	TE	ST	TE	ST	TE	ST	TE	ST	TE	ST	TE	ST	TE	ST
10		2.0 ± 0.0	37.4 ± 0.7											
15	2.0 ± 0.1	7.4 ± 1.8	5.8 ± 0.1	5.6 ± 0.3	5.1 ± 0.2	3.3 ± 0.2	7.0 ± 0.1	4.6 ± 0.2	16.7 ± 0.4	13.4 ± 0.8	10.7 ± 0.3	8.7 ± 0.6	36.8 ± 0.5	35 ± 0.9
25	3.8 ± 0.4	4.5 ± 0.7	2.5 ± 0.1	2.1 ± 0.2	2.0 ± 0.1	1.2 ± 0.2	2.4 ± 0.1	1.2 ± 0.1	5.0 ± 0.1	3.3 ± 0.2	3.5 ± 0.1	2.7 ± 0.1	12.7 ± 0.2	10.4 ± 0.7
30	3.2 ± 0.3	6.7 ± 0.7	2.0 ± 0.0	1.4 ± 0.1	1.1 ± 0.0	1.3 ± 0.1	1.4 ± 0.1	1.4 ± 0.2	3.5 ± 0.1	3.0 ± 0.3	1.9 ± 0.0	1.9 ± 0.1	7.6 ± 0.1	8.8 ± 0.6

**Table 3:** Temperate *Ae. albopictus* adult longevity. Longevity (days) of *Ae. albopictus* temperate adult females maintained at four constant temperatures: 10, 15, 25, and 30 °C.

T (°C)	Mean ± SE (Days)	Range (min–max) (Days)
10	5.7 ± 0.4	2–10
15	27.9 ± 2.0	8–45
25	64.5 ± 3.8	32–92
30	53.5 ± 4.7	14–86

**Table 4:** Temperate *Ae. albopictus* fecundity. Duration of gonotrophic cycle (mean and standard error in days) and number of eggs (mean and standard error) for temperate *Ae. albopictus* at 25 and 30 °C.

	25 °C				30 °C			
	n	Duration (Mean ± SE)	Minimum Duration	Number of Eggs (Mean ± SE)	n	Mean	Minimum Duration	Number of Eggs (Mean ± SE)
Cycle 1	20	4.2 ± 0.1	4	31.7 ± 2.0	20	3.1 ± 0.1	2	55.9 ± 4.0
Cycle 2	20	4.0 ± 0.0	3	40.1 ± 2.3	12	3.3 ± 0.2	2	55.0 ± 8.2
Cycle 3	20	3.0 ± 0.0	3	40.8 ± 2.1	3	4.3 ± 0.9	3	60.7 ± 13.2
Cycle 4	20	4.0 ± 0.0	4	40.4 ± 2.3				
Cycle 5	17	3.0 ± 0.0	3	39.4 ± 2.5				
Cycle 6	16	3.6 ± 0.2	3	31.6 ± 4.7				
Cycle 7	16	3.9 ± 0.1	3	30.0 ± 3.5				
Cycle 8	13	4.2 ± 0.4	3	25.4 ± 5.0				
Cycle 9	9	4.1 ± 0.1	4	29.4 ± 8.3				
Cycle 10	5	4.2 ± 0.2	4	19.0 ± 0.4				
Cycle 11	2	7.5 ± 2.5	5	6.0 ± 3.0				
Mean		3.8 ± 0.1		34.3 ± 1.2		3.3 ± 0.1		56.0 ± 3.7

**Figure A1:**
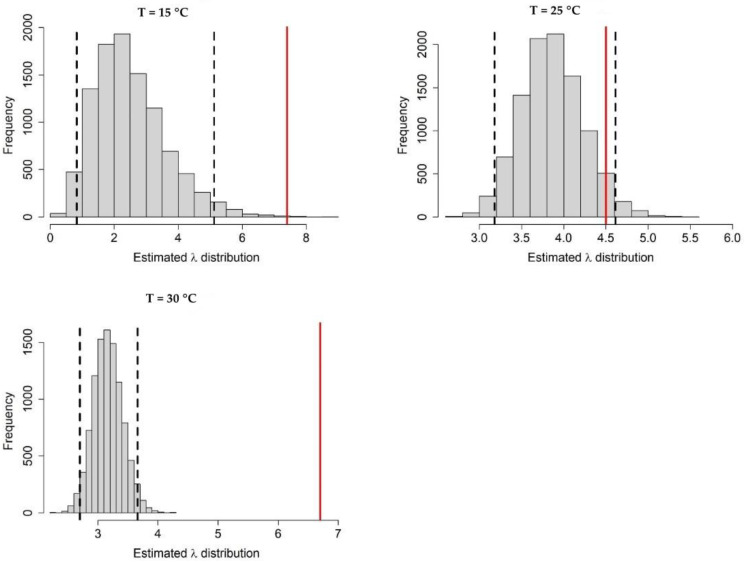
Estimated posterior distributions of Λ_E_(T), the average length of time (days) between immersion of eggs in water and hatching response at 15, 25, and 30 °C. Dashed black lines represent the 2.5 and 97.5 quantiles. Solid red lines represent the average value for subtropical *Ae. albopictus*.

**Figure A2:**
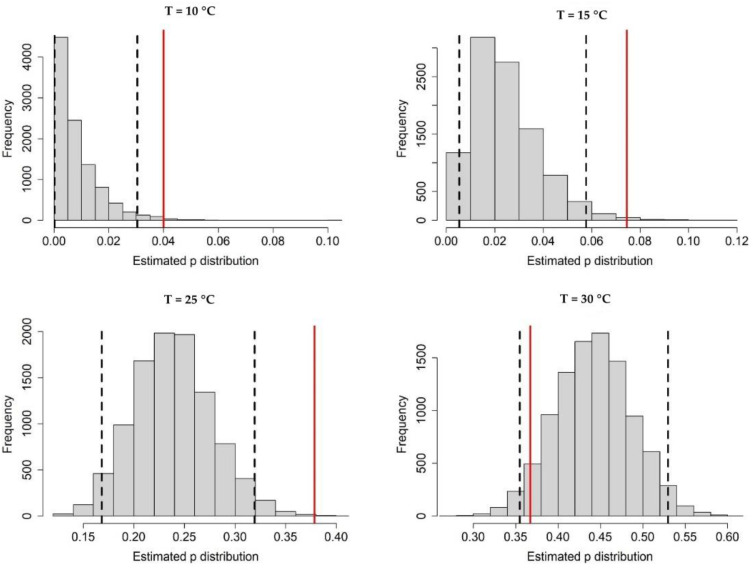
Estimated posterior distributions of P_E_(T), the egg hatching rate at 10, 15, 25, and 30 °C. Dashed black lines represent the 2.5 and 97.5 quantiles. Solid red lines represent the average value for subtropical *Ae. albopictus*.

**Figure A3:**
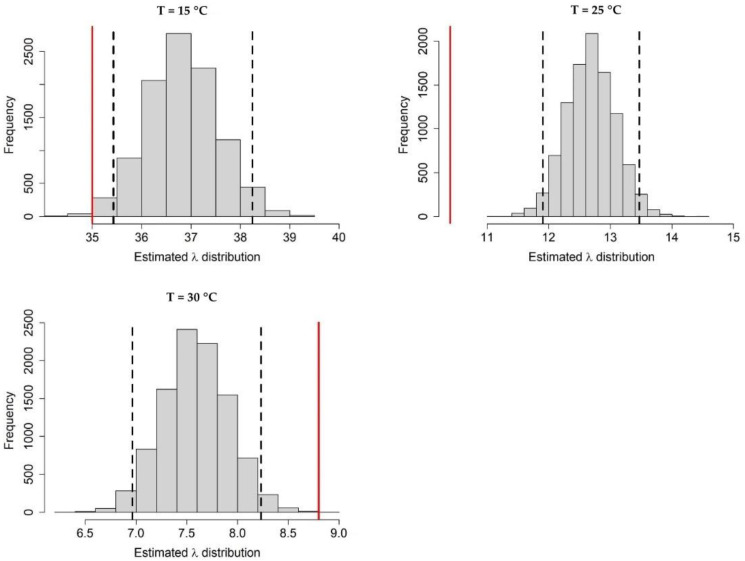
Estimated posterior distributions of Λ_L1-A_(T), the average duration (days) of development from L1 to A at 15, 25, and 30 °C. Dashed black lines represent the 2.5 and 97.5 quantiles. Solid red lines represent the average value for subtropical *Ae. albopictus*.

**Figure A4:**
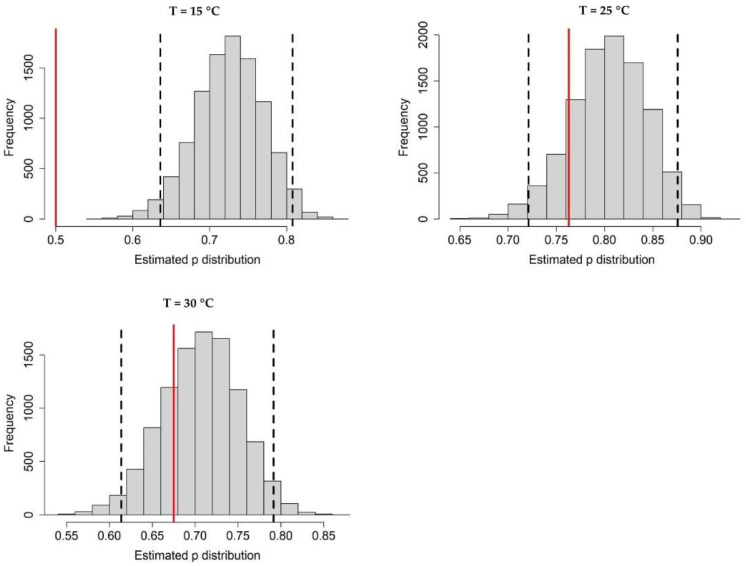
Estimated posterior distributions of P_L1-A_(T), the survival rate from L1 to A at 15, 25, and 30 °C. Dashed black lines represent the 2.5 and 97.5 quantiles. Solid red lines represent the average value for subtropical *Ae. albopictus*.

**Figure A5:**
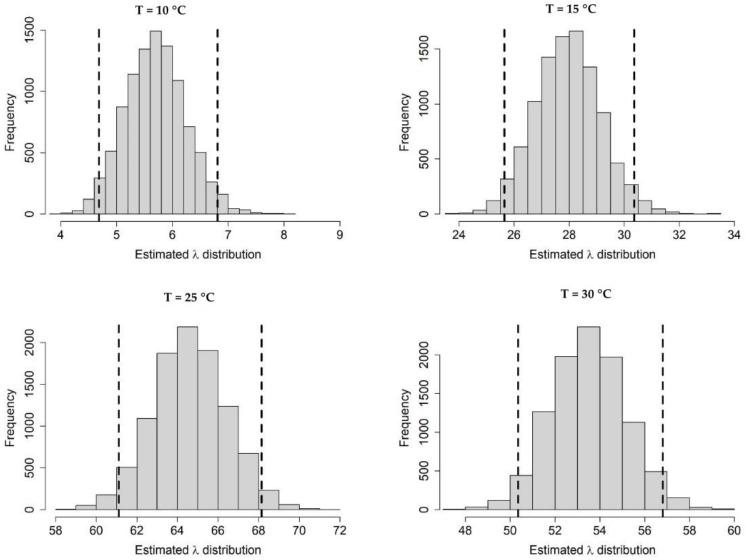
Estimated posterior distributions of Λ_A_(T), the average adult female longevity (days) at 10, 15, 25, and 30 °C. Dashed black lines represent the 2.5 and 97.5 quantiles.

**Figure A6:**
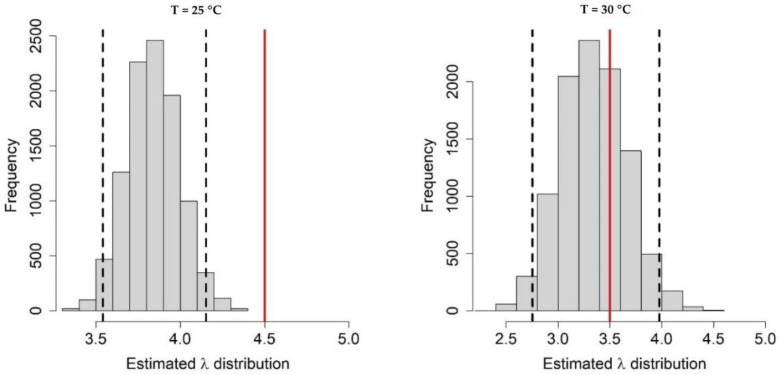
Estimated posterior distributions of Λ_G_(T), the average duration of gonotrophic cycle at 25 and 30 °C. Dashed black lines represent the 2.5 and 97.5 quantiles. Solid red lines represent the average value for subtropical *Ae. albopictus*.

**Figure A7:**
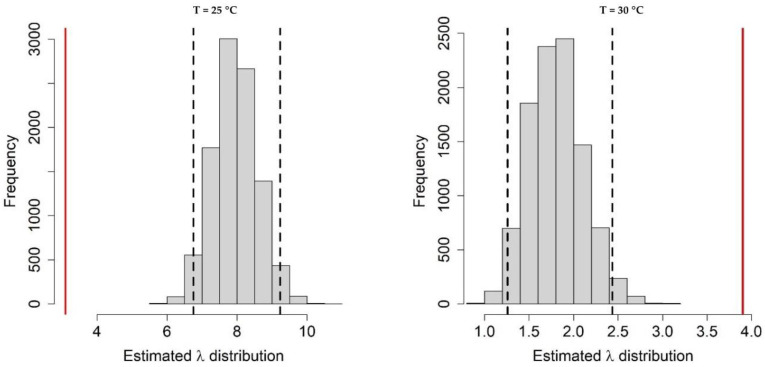
Estimated posterior distributions of Λ_NG_(T), the number of gonotrophic cycles completed on average by a female mosquito at 25 and 30 °C. Dashed black lines represent the 2.5 and 97.5 quantiles. Solid red lines represent the average value for subtropical *Ae. albopictus*.

**Figure A8:**
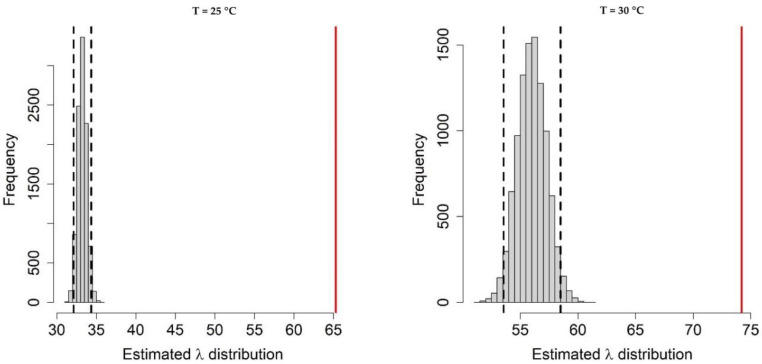
Estimated posterior distributions of Λ_NE_(T), the number of eggs laid on average by a female mosquito at 25 and 30 °C for each gonotrophic cycle. Dashed black lines represent the 2.5 and 97.5 quantiles. Solid red lines represent the average value for subtropical *Ae. albopictus*.

**Figure A9:**
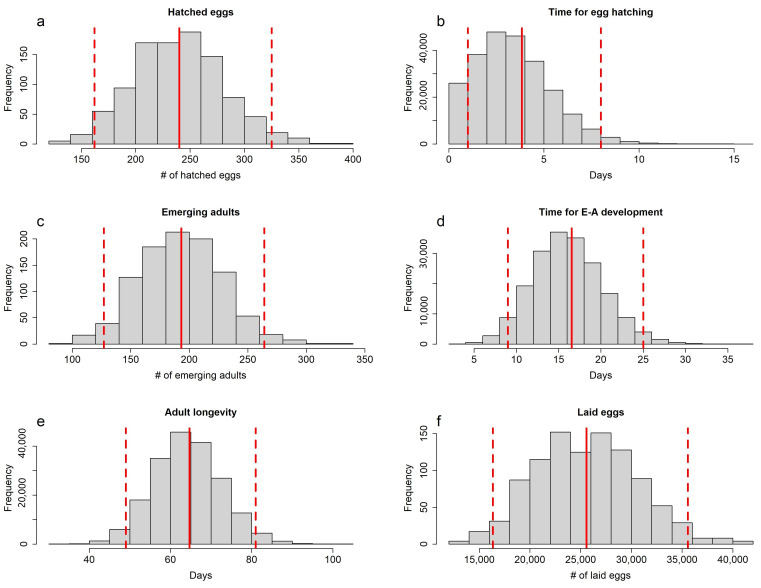
Distributions of quantities of interest for simulated (1000 simulations) life history of 1000 initial eggs at 25 °C. (**a**) Number of hatched eggs; (**b**) time for egg development; (**c**) number of emerging adults; (**d**) time of development from the egg stage; (**e**) adult longevity; (**f**) total number of eggs laid by all females. Dashed red lines represent the 2.5 and 97.5 quantiles, solid red lines represent the average value.

**Figure A10:**
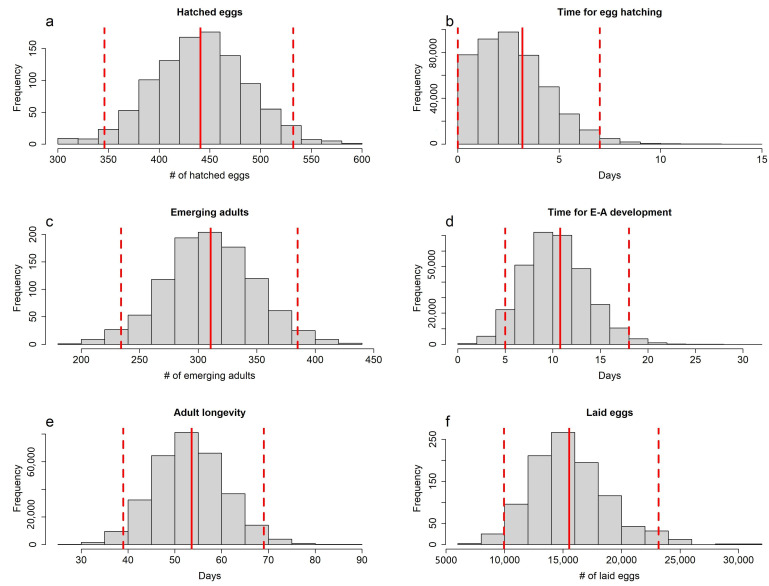
Distributions of quantities of interest for simulated (1000 simulations) life history of 1000 initial eggs at 30 °C. (**a**) Number of hatched eggs; (**b**) time for egg development; (**c**) number of emerging adults; (**d**) time of development from the egg stage; (**e**) adult longevity; (**f**) total number of eggs laid by all females. Dashed red lines represent the 2.5 and 97.5 quantiles, solid red lines represent the average value.

## Appendix A

### Appendix A.1. Bayesian Fitting of the Posterior Distributions of Life History Parameters

We applied a Bayesian approach by fitting through a Markov Chain Monte Carlo technique a Poisson distribution for developmental times, longevity, number of laid eggs, number and length of the gonotrophic cycles, and a binomial distribution for survival rates.

For instance, for each temperature T∈{10, 15, 25, 30 °C}, we had a set of observed duration of development from L1 to A *τ_L_*_1-*A*_(*T*)*_i_*, *i* = 1,…*k*(*T*), where *k*(*T*) is the number of emerging adults, which is different across the tested temperatures. Thus, the likelihood of recording our observations assuming they belong to a Poisson distribution with average *λ* is

(A1) $$L=\prod_{i=1}^{k\left(T\right)}e^{-\tau_{L1-A}{\left(T\right)}_{i}}\cdot \frac{\tau_{L1-A}{\left(T\right)}_{i}{}^{\lambda }}{\lambda !}\;.$$

Recalling that such experiment was conducted using N cups with *n* initial L1 specimens, we have $k\left(T\right)=\overset{N}{\underset{i=1}{\sum }}k_{i}\left(T\right)$, where *k_i_*(*T*) is the number of emerging adults in cup *i*. Thus, the likelihood of observing the recorded survivals assuming they belong to a binomial distribution with probability *p* is

(A2) $$L=\prod_{i=1}^{N}\left(\begin{array}{c} n\\ k_{i}\left(T\right) \end{array}\right)p^{k_{i}\left(T\right)}{\left(1-p\right)}^{n-k_{i}\left(T\right)}.$$

The posterior distributions of λ and *p*, denoted by Λ and P respectively, were obtained by using random-walk Metropolis–Hastings sampling approach and normal jump distributions [38]. A total of 100,000 iterations were performed and a burn-in period of 5000 steps was chosen.

Afterwards, if for instance, we wanted to test whether the average duration of development from L1 to A at 25 and 30 °C were substantially different, we randomly extracted 10,000 values from the two respective posterior distributions Λ_L1-A_(25) and Λ_L1-A_(30), computed the differences x_i_, i.e., x_i_ = (λ_i_(30) − λ_i_(25))_i = 1, …, 10,000_, and evaluated the probability for the two distributions to be similar as

(A3) q=min(P(x≤0),1−P(x≤0)).

If *q* < 0.05, we considered the two distributions to be statistically different.

Finally, to assess if the average values reported for subtropical *Ae. albopictus* in [18] are significantly different from our estimates, we estimated the probability of observing the subtropical values given our results. For instance, if the average duration of development from L1 to A at 25 °C was *s* days for subtropical mosquitoes, we evaluated the probability of observing the subtropical value *s* given our posterior distribution Λ_L1-A_(25) as

(A4) q=min(P(λ≤s), 1−P(λ≤s)).

If *q* < 0.05, we considered the two estimates to be statistically different.

The estimated posterior distributions for some quantity of interest are shown in Figure A1, Figure A2, Figure A3, Figure A4, Figure A5, Figure A6, Figure A7 and Figure A8, together with the available average observations for subtropical *Ae. albopictus* as reported in [18].

### Appendix A.2. Adult Survival

We modeled adult survival *S* as age dependent according to a Weibull function

(A5) $$S\left(t\right)=\exp\left(-\frac{a}{g}\cdot t^{g}\right)$$

where *t* is the age and *a* and *g* are free parameters estimated by a least-squares method.

### Appendix A.3. Population Dynamics Simulation

We simulated the life history of 1000 temperate initial eggs at 25 and 30 °C according to our estimates. More specifically, we run 1000 simulations for each temperature T∈{25,30}, and for each of them, we drew the number of hatching eggs *e* and the time of emergence from P_E_(T) and Λ_E_(T), respectively. Subsequently, the number of emerging adults *a* (*a* ≤ *e*) and their time of development was drawn from P_L1-A_(T) and Λ_L1-A_(T). Finally, *a*/2 (only females) adults laid *n* eggs for each gonotrophic cycle *c*, with *n* and *c* drawn from Λ_NE_(T) and Λ_NG_(T) respectively, and they live for *l* days, with *l* extracted from Λ_A_(T).

Figure A9 and Figure A10 show the distributions, for the 1000 simulations, of the above-mentioned quantities of interest.
